# Neural Design Principles for Subjective Experience: Implications for Insects

**DOI:** 10.3389/fnbeh.2021.658037

**Published:** 2021-05-05

**Authors:** Brian Key, Oressia Zalucki, Deborah J. Brown

**Affiliations:** ^1^School of Biomedical Sciences, The University of Queensland, Brisbane, QLD, Australia; ^2^School of Historical and Philosophical Inquiry, The University of Queensland, Brisbane, QLD, Australia

**Keywords:** sentience, awareness, feeling, qualia, consciousness, *Drosophila*, pain

## Abstract

How subjective experience is realized in nervous systems remains one of the great challenges in the natural sciences. An answer to this question should resolve debate about which animals are capable of subjective experience. We contend that subjective experience of sensory stimuli is dependent on the brain’s awareness of its internal neural processing of these stimuli. This premise is supported by empirical evidence demonstrating that disruption to either processing streams or awareness states perturb subjective experience. Given that the brain must predict the nature of sensory stimuli, we reason that conscious awareness is itself dependent on predictions generated by hierarchically organized forward models of the organism’s internal sensory processing. The operation of these forward models requires a specialized neural architecture and hence any nervous system lacking this architecture is unable to subjectively experience sensory stimuli. This approach removes difficulties associated with extrapolations from behavioral and brain homologies typically employed in addressing whether an animal can feel. Using nociception as a model sensation, we show here that the *Drosophila* brain lacks the required internal neural connectivity to implement the computations required of hierarchical forward models. Consequently, we conclude that *Drosophila*, and those insects with similar neuroanatomy, do not subjectively experience noxious stimuli and therefore cannot feel pain.

## Introduction

Where in the phylogenetic tree of life do organisms evolve as sentient creatures with subjective experience of sensory stimuli? Given that non-human animals lack the capacity for verbal report, we must resort to making inferences about the nature of their experience based upon similarities between their behavioral responses, neural architectures (structures) and neurophysiological processes (functions) and those of humans. Here we face a quandary. What criteria do we base our decisions on? Do we treat the various criteria just cited—behavioral, structural, functional—as conjointly necessary or as an inclusive disjunction? There are those who, for example, accept that any similarity in noxious behavior in animals and humans constitutes evidence for the presence of neural structures generating subjective experience. There is no reason, however, to accept this naïve premise. We contend that a better strategy is, first, to identify neural computations that are necessary for subjective experience such as in the case of pain, and then to determine which animals have the neural architectures necessary to execute those algorithms (Key and Brown, 2018; Brown and Key, 2021).

Our approach is built on two contestable premises. First, that neural processing is necessary for subjective experience. While this may seem scientifically reasonable given the wealth of experimental and clinical data on this matter, there are those that propose that no amount of physical knowledge about the nervous system will ever lead to an understanding of subjective experience (Jackson, 1982). Others instead envision subjective experience as an intrinsic property of sensory states (i.e., neural activity associated with sensory stimuli) (Tye, 2015). If one adopts this latter point of view, then the mere possession a nervous system is sufficient for subjective experience. The second premise is that specific types of neural computations (i.e., informational processing) are needed for subjective experience. We build on the universal biological principle that structure-determines-function. For instance, subjective experience necessarily requires the presence of some biophysical mechanism that converts a sensory input into neural activity. Should the structural basis of that mechanism be perturbed then downstream functions will be altered. Nature has conveniently presented us with some clear examples of the structure-determines-function principle with respect to the subjective experience of pain. There are some animals that have evolved genetically altered ion channels that prevent their sensory neurons from responding to select noxious stimuli and, as a result, they are said to be insensitive to pain. For example, the blue-ringed octopus fails to respond to its own venom while the naked-mole rat and its close relatives are insensitive to the harmful effects of acid (Flachsenberger and Kerr, 1985; Smith et al., 2011; Zakon, 2012). Following this reasoning, we propose that specific neural circuits are needed to execute neural computations necessary for subjective experience. As a consequence, animals lacking the appropriate neural circuitry will be unable to subjectively experience—just as non-human primates lacking the neural circuitry linking vocalization and mandibular oscillations fail to produce speech (Brown et al., 2021). The challenge we face is to identify the neural circuitry that is necessary for subjective experience. While we do not pretend that this solves the “hard problem” of consciousness (Chalmers, 1995), it does afford minimal criteria for deciding which species have at least the capacity for subjective experience. We will use pain here for illustrative purposes only; the same principles of reasoning apply when assessing a species’ capacity for other modes of conscious experience.

Addressing the computational bases of consciousness (Reggia et al., 2016; Dehaene et al., 2017; Cleeremans et al., 2020; Rolls, 2020; Wiese, 2020) is gaining momentum and proving more insightful than attempts to define and adopt gross behavioral roles of consciousness to understand subjective experience (Seth, 2009). Unfortunately, uncertainty surrounding the nature of neural architectures generating subjective experience in the human brain has confused the debate about animal subjective experience. For example, suggestions that subjective consciousness of pain arises at the level of the midbrain (Merker, 2016) have been leveraged in support of the conclusion that insects experience pain. Since insects supposedly contain brain regions capable of performing similar neural computations to those of midbrain regions, it is assumed that they also have the potential to feel pain (Barron and Klein, 2016). Yet, there has been little interrogation of the core assumptions underlying the claim that subjective consciousness can arise in the midbrain. We return to this matter in Sections “Do Insects Really Feel With Their Analogous “Midbrain” Structures?” and “Are Mental Maps a Necessary Condition for Subjective Experience?”.

We present here a framework based on a hierarchical forward models algorithm (Key and Brown, 2018) for addressing the question of subjective experience in insects, in opposition to claims that these animals have feelings (Allen-Hermanson, 2016; Key, 2016c; Key et al., 2016; Schilling and Cruse, 2016; Adamo, 2019). In Section “Cerebral Cortex As the Seat of Human Subjective Experience,” we clarify the meaning of subjective terminology that we deploy in this article, much of which aligns with standard philosophical usage, and briefly highlight some of the major historical observations that ground the premise that subjective consciousness in humans is dependent on the cerebral cortex. These early studies expose the dangers of anthropomorphism and casual anecdotal observations. That the cerebral cortex is necessary for human subjective experience is significant—not because we believe that this or that brain region is necessary for consciousness in any animal—but rather because the cerebral cortex provides insight into the types of neural computations generating subjective experience. Ultimately, the debate has to proceed on the basis of analogies at the level of information-processing tasks, not at the level of homologous gross similarities in brain regions, and it is partly for this reason that we find the evidence in favur of insect sentience underwhelming. We discuss in Section “Structure-Determines-Function Principles Underlying Neural Design” the cardinal principles of brain function that predicate our choice of neural computations underpinning subjective experience. In Section “A Hierarchical Forward Models Framework for Subjective Experience,” our neural architectural framework for subjective experience is presented. Using nociception as a sensory model, we assess in Section “Do Insects Possess the Neural Architecture Necessary for Pain?” whether, according to our framework, the insect nervous system has the neural circuitry required for subjective experience. Finally, we conclude in Section “Concluding Remarks” that there is insufficient evidence to support the conclusion that insects are capable of subjective experience.

To be clear, we are not purporting to be able here to prove categorically that insects are incapable of subjective experience. Our argument is directed rather at those who claim to have sufficient evidence to reject the null hypothesis that says insects lack subjective experience. We offer our model in support of the null hypothesis in the realization that the model may be deficient and/or that our structural understanding of the insect nervous system may be incomplete. Nonetheless, we believe that the structure-determines-function based approach adopted here remains the most plausible way forward to addressing the ‘other minds’ problem (Harnad, 1991).

## Cerebral Cortex as the Seat of Human Subjective Experience

### Terms of Reference

To avoid confusion, let us begin by clarifying how we intend to use subjectivist terminology. Following standard philosophical usage (Nagel, 1974), we define subjective experience of sensory stimuli as referring to the ‘what-it-is-likeness’ of a conscious state—a state also referred to as subjective awareness, subjective consciousness, qualia (Jackson, 1982), phenomenal (‘P’) consciousness (Block, 1995), or simply, feelings. According to this terminology, if there is nothing it feels like to be in a given state—e.g., when a noxious stimulus is administered but not felt after an injection of local anesthetic—then it is not subjective experience. We then distinguish between sentience—the subjective awareness of sensory processing—and consciousness more generally. While these terms are sometimes used interchangeably, it is possible to be consciously aware of something (e.g., a thought) without a corresponding sensory feeling, and, presumably, vice versa. However, when one is consciously aware of a sensory stimulus, it necessarily feels like something, and this, following standard usage, is called being sentient. Because verbal report is currently the only valid means of assessing whether a sensory stimulus feels like something, humans are, by default, the gold standard for understanding these experiences. The task for the comparative neurobiologist is to determine the criteria for extending attributions of ‘sentience’ to non-human species.

Awareness is the defining feature of subjective experience—without awareness there can be no subjective experience. To be clear, when we refer to ‘awareness,’ we do not mean ‘access consciousness’ in Block’s (1995) terminology. Access consciousness is defined functionally, not qualitatively—that is, in terms of information that is available to the executive system for use in reporting, reasoning and rationally guiding action (ibid, p. 228). On Block’s construal, access consciousness can be dissociated from phenomenal consciousness—there may be no particular quality to the process by which the contents of consciousness are accessed and what we do experience can subjectively exceed that which we can report. This latter observation—part of what Block later develops into his ‘overflow’ argument (Block, 2011)—suggests that the extent to which access consciousness to sensory information can be revealed in self-reports is due to the fact that it involves conscious reflection on subjective experience, a kind of metalevel self-awareness.

We support the view that subjective experience is automatic and does not depend on meta-cognition. It does, however, depend on awareness being conscious. We, like others before us (Davis, 1982; McClelland, 2013), deny claims that subjective experience is possible without conscious awareness—there are no ‘unfelt feelings’ or ‘absent qualia’ that warrant being thought about as instances of consciousness rather than as neurophysiological or information-processing events that occur below the threshold of consciousness. In the example of pain, it is a neurophysiological misunderstanding to refer to ‘unfelt’ pain (Palmer, 1975; Reuter and Sytsma, 2020). Failure to recognize this leads to a conflation between terms for conscious experiences and terms for non-conscious processing. There is another term for non-conscious processing of noxious stimuli—*nociception* (Sherrington, 1906)—and references to ‘unfelt pains’ blur this crucially important distinction. The pain/nociception distinction lies at the heart of our approach to subjective consciousness in animals. That conscious pain and non-conscious nociception involve different neural processing operations is what enables researchers to make informed judgments about a species’ capacity for pain or lack thereof. An animal’s lack of the necessary pain mechanisms is the basis for legitimately inferring that it cannot feel pain (Brown and Key, 2021).

### The Discovery of a “Feeling” Cerebral Cortex

Many prominent naturalists, physiologists and biologists in the nineteenth century earnestly sought to determine whether animals possess subjective experience. There are lessons to be learnt from this work, particularly with respect to recognizing the shortcomings of simple narratives or just-so stories and avoiding the pitfalls of anthropomorphism. In the mid-19th century, the French physician, Marie Jean Pierre Flourens, heralded a new age of experimental physiology by systematically assessing the effects of lesions and external stimulation of the nervous system on the behavior of vertebrate animals (Flourens, 1842). He revealed that distinct regions of the nervous system have distinct functions (ibid, pp. 235–237) and he was an early advocate of the now well-established principle in biology that structure-determines-function (ibid, p. 22). Flourens pioneered a methodical approach to identifying neural regions necessary for behaviors by lesioning specific neural tissues. Based on a series of experimental manipulations and perturbations in several vertebrate species, Flourens concluded that the cerebral lobes are the site of perception of sensation (perception being the feeling and sensation being the neural processing of stimuli) as well as the source of will and intelligence. Thus, he revealed that the nervous system is not a homogenous tissue and that different neural structures have distinct functions (ibid, p. 56).

Interestingly, the idea of the cerebral localization of perception was initially treated with some skepticism. Pflüger (1853) published his work on the sensory functions of the spinal cord of vertebrate animals. Pflüger (1853, p. 12) could not imagine how any behavior exhibited by headless animals could occur without some form of perception. For instance, he observed that a decapitated frog would make “restless” movements with its trunk and limbs which he attributed to feelings of “discomfort” (ibid, p. 15). Further, he noted that if the paw of a headless frog was pulled, then the animal would hide the paw under his stomach and crouch in fear (ibid, p. 16). The use of intentional language permeates Pflüeger’s causal understanding of animal behavior. Pflüeger (ibid, pp. 24–25) adopted the view that when both a normal animal and a headless animal responded similarly to the heat of fire on their skin, then pain must arise below the level of the neural lesion. He believed that motor behaviors were mostly driven at the level of the hindbrain and below. While these results were similar to those of Flourens, Pflüeger’s interpretation of their significance was markedly different. Pflüeger was, in contrast to Flourens, an advocate of motor behaviors as an indicator of feelings in animals. Based merely on his intuitions about the source of headless frog behaviors, Pflüeger dismissed Flourens’ idea that perception arises in the cerebral lobes.

Not long after Pflüerger’s work, Goltz (1869) sought to locate “the seat of the soul” (ibid, p. 54), the physiological basis of cognition and consciousness as that expression would have been understood at the time, by examining what happened to the behavior of frogs when different brain regions were removed. Goltz removed the frog forebrain and found that these animals required forcible feeding since they no longer responded to the sight of prey (even when they were starved of food beforehand) (ibid, p. 56). They also lacked any spontaneous movement—which he referred to in a loaded fashion as “voluntary or spontaneous” (ibid, p. 60)—and instead found that they remained fixed and motionless for long periods. He concluded (as Flourens had for mammals) that the forebrain in the frog was responsible for mental processing. Goltz realized that feelings were subjective and could only be experienced by an organism that was the subject of a sensory stimulus (ibid, p. 127). The experimenter is limited merely to observation of the resultant movements produced by the animal and, as such, lacks any knowledge about an animal’s inner experience. He noted that, at best, one could only assume that in the absence of any behavioral response to a sensory stimulus, an animal probably lacked the corresponding subjective experience. Thus, Goltz reached the striking conclusion that one could only infer that animals not responding to sensory stimuli do not feel these stimuli, and that nothing could be concluded about conscious experience with respect to animals exhibiting such behavioral responses.

Nonetheless, even to this day there are some researchers who continue to conclude that certain behaviors associated with subjective experience in humans are sufficient indicators of subjective experience in non-human animals (Birch et al., 2020). Even though an insect—like a human—can behave in a select way to a sensory stimulus, it does not follow of necessity that both insect and human subjectively experience that sensory stimulus. The work of both Flourens and Goltz were foundational in establishing two important premises: first, that the cerebral lobes are the site of perception in non-human mammals, and second, that animal behaviors do not necessarily reflect the presence of subjective experience. However, there is tension between these premises since the ability to localize perception to the cerebral lobes was reliant on the assumption that the observed behaviors were indeed reflective of subjective experience. This conflict was not resolved until experimental observations progressed from non-human animals to non-human primates, and then finally to humans, who can verbally report their subjective experience.

During the remaining 19th century, evidence emerged to further support the idea that discrete functions are localized to specific regions of the mammalian cerebral cortex (Ferrier, 1873, 1876). These discoveries grounded the principle that *structure-determines-function* in the brain. They also heralded a new era of experimental neurophysiology in the early 20th century using techniques that were subsequently applied in non-human primates (Sherrington, 1906), and then ultimately to humans during neurosurgery (Cushing, 1909; Penfield, 1958, 1959). It was these latter clinical interventions, involving direct electrical stimulation of the cerebral cortex in awake patients, that provided clear and demonstrable evidence for the role of the cerebral cortex in subjective experience. The first half of the 20th century also saw a plethora of clinical reports of cortical injuries causing discrete loss of sensory function arising from physical lesions sustained during the great wars as well as from disease states (Russel and Horsley, 1906; Wilson, 1927; Russell, 1945; Marshall, 1951). The absence of feeling resulting from cortical damage, for example, from the shrapnel wounds of returned WWII soldiers, lent particular credence to the idea that cortical activity is necessary for subjectivity (Marshall, 1951).

### Do Insects Really Feel With Their Analogous “Midbrain” Structures?

Despite strong evidence for the current consensus view in neuroscience that the cerebral cortex is necessary for subjective experience in humans (Key, 2015, 2016a,c; Laureys et al., 2015; Boly et al., 2017; Odegaard et al., 2017; Key and Brown, 2018; Mashour, 2018; Graziano et al., 2019; Lamme, 2020; LeDoux, 2020a,b; Rolls, 2020), some past overreaching arguments about the subcortical location of feelings remain influential. Merker (2007) initially proposed that sentience arose subcortically (unfortunately using unorthodox neuroanatomical terminology) and he particularly defended this thesis with evidence of motor behaviors of decorticate rats reported by Woods (1964) and of humans with hydranencephaly (see more about this below). Merker (2016) later reported that the rats lesioned by Woods (1964) were actually mesencephalic animals and that the midbrain was therefore necessary and sufficient for consciousness. While Barron and Klein (2016) adopted some of Merker’s original subcortical terminology (which consisted of the midbrain and a mix of other nuclei) they specifically argued the case for insect consciousness on the grounds that the insect brain supposedly contains regions functionally analogous to the vertebrate midbrain. For instance, they noted that the insect central complex performs functions analogous to the “vertebrate tectum/colliculus” (ibid, p. 4903), which is part of the vertebrate midbrain.

Merker’s argument, however, quickly unravels when it is subjected to experimental cross-examination. Removal of the visual midbrain (superior colliculus) in rodents and primates has only modest effects on visual consciousness (Albano et al., 1982; Milner et al., 1984). If the superior colliculus were indeed necessary for visual consciousness, animals would be rendered phenomenally blind by such a lesion, yet they are not. Notably, it is lesions to the visual (V1) cortex in humans that instead lead to blindness (Holmes, 1918). Moreover, lesions to select cortical regions downstream of V1 (e.g., V4 and ventral occipitotemporal cortex) cause impaired visual color experience (achromatopsia) in humans (Zeki, 1990).

Merker claims that the midbrain is also sufficient for subjective experience since hydranencephalic children born with substantial (although not complete) loss of cerebral tissue are able to respond to some sensory stimuli. Interestingly, Sherrington (1906, pp. 254) had noted much earlier that many behavioral responses are reflexive and could be elicited in unconscious decerebrate animals, in children lacking both midbrain and cortex, and in normal adults during ether anesthesia. Rare reports of hydrocephalic individuals living seemingly normal lives (Feuillet et al., 2007) have sometimes been used as evidence that the cortex is not necessary for subjective experience (Doerig et al., 2020). However, what further investigation of these reports reveal is that the cortex exhibits considerable plasticity especially when the insults occur early in life (Herbet and Duffau, 2020). One also has to be cautious of reports on these rare cases since quantitative analyses have revealed that hydrocephalus does not necessarily cause substantial loss of cortical tissue and where some cortical tissue remains, so too does there remain some degree of functionality (Chen et al., 2017; Alders et al., 2018; Ferris et al., 2019).

While Merker (2007) did not quantify cortical loss in hydranencephaly he noted that, in general, these children can have “variable remnants of cortex” including inferiomedial occipital, basal portions of temporal cortex, and “midline cortical tissue along the falx extending into the medial frontal cortex.” Given that most hydranencephalic children are moribund and die during infancy (Hoffman and Liss, 1969; Bae et al., 2008) it is imperative that brain neuroimaging data is correlated with sensory testing. While Merker (2007) recorded his “impressions” about the behaviors displayed by five hydranencephalic children he unfortunately neither analyzed cortical images nor undertook quantitative sensory/behavioral testing on this rather small cohort of children. Thus, Merker’s necessity and sufficiency claims for the subcortical basis of subjective experience remain unsubstantiated and are insufficient to ground claims of subjective experience in insects.

### Are Mental Maps a Necessary Condition for Subjective Experience?

Feinberg and Mallatt (2020) have proposed that subjective experience emerged around 580 to 520 million years ago within invertebrate animals. They note that this hypothesis critically depends on “two fundamental assumptions.” First, animals will subjectively experience sensory stimuli (such as vision and hearing) if they possess neural pathways that create “mental maps” of different sensory stimuli which converge into a unified, multisensory “image” of the environment. Second, animals must be capable of complex operant learning (i.e., “learning and remembering from experience to avoid harmful stimuli and to approach helpful stimuli”). Since insects meet these two criteria they are *ipso facto* considered by Feinberg and Mallatt to be capable of subjective experience. It is not our intention here to discuss any potential shortcomings with these two assumptions in detail, as those arguments have been presented elsewhere (Key, 2015, 2016b,d; Key and Brown, 2018). We have already cautioned above about the dangers of relying on behaviors (in this case, operant conditioning) as implying subjective awareness. However, we draw attention to some salient points. There is nothing in the possession of sensory maps or so-called ‘images’ that necessitates subjective experience. For example, converging sensory maps of different somatosensory modalities are present in the non-feeling spinal cord of mammals (Gradwell and Abraira, 2021) and in the non-feeling midbrain tectum of fish (Hiramoto and Cline, 2009; Key, 2016d).

Feinberg and Mallatt borrow the term ‘image’ from Damasio who uses it to represent an integrated, topographic sensory map (Damasio, 2012; Damasio and Carvalho, 2013). Damasio and Carvalho claim that sensory maps associated with both interoception and exteroception are essential for subjective experience. This antecedent condition is presented and accepted without question. Damasio and Carvalho (2013) then reasoned that those subcortical regions lacking sensory maps (e.g., the amygdalus) could not be involved in generating feelings. These authors noted that the brainstem and cortex were the principal brain regions in humans containing sensory maps but they concluded that the brainstem, and not the cortex, was responsible for feelings. This conclusion rested heavily on Merker’s descriptions of hydranencephalic children (see section “Do Insects Really Feel With Their Analogous “Midbrain” Structures?”) and followed Damasio’s earlier conclusion that subjective experience of both vision and hearing was also occurring in the midbrain tectum of these children (Damasio, 2012, pp. 80–83).

What has emerged from the ideas of Merker, Feinberg, Mallatt, and Damasio is that careful consideration ought to be given to assessing the nature and origins of the assumptions and intuitions that underpin the viewpoint that the cortex is not the neural substrate for subjective experience. This need for critical reflection has certainly guided our short historical account throughout this section. It is now time to address the most puzzling question in the natural sciences: what are the neural computations executed by the cerebral cortex that generate subjective experience? In the next section, we describe some fundamental neural design principles that have helped us begin to address this perplexing question.

## Structure-Determines-Function Principles Underlying Neural Design

### Basic Design Features Governing Neural Function

Given the ease with which we subjectively experience the world—just close and open your eyelids to experience the seemingly instantaneous percept of a rich visual field—it is too easily forgotten that the nervous system must perform complex neural computations at multiple structural levels (retina, thalamus, and cortex) to generate this experience. Let’s take a closer look at these computations, using vision as an example. We use vision here simply because the sheer volume of empirical studies of this faculty (particularly in humans) has revealed the fundamental computational principles of sensory systems across diverse species. What we have learnt from the visual system has proven to be pertinent to other sensory modalities as well as to nociception/pain. We will return to pain in the Section “A Hierarchical Forward Models Framework for Subjective Experience” because this subjective experience has been historically instrumental in understanding both the nature and evolution of consciousness (as discussed in Section “Cerebral Cortex As the Seat of Human Subjective Experience”).

The only source of visual information that the brain receives about the visual environment comes via retinal ganglion neurons (RGNs). Each RGN is uniquely responsive to light falling upon a restricted circular domain in the retina (called a receptive field). Light falling within this domain specifically affects the neuronal firing of that RGN. Each RGN is a separate monitoring device in the retina—there are no downstream neurons in the retina that integrate across the retinal surface. The retina is therefore blind to overall object shape. The only information exiting the retina is the train of independently firing sequences from each RGN. Consequently, it is the brain that must perform the computations necessary to infer the nature of the visual stimulus arising from multiple small detectors in the retina. Despite its oversimplification, this demonstration of visual coding exposes three important design principles of nervous systems in general:

(1) *Function arises from neural computations performed by specific circuits.* The connectivity of neurons within circuits is critical for their function.
(2) *Neural processing is hierarchically organized.* Environmental stimuli are processed in stages to progressively reveal more and more features. For example, within the retina, photoreceptors respond to the presence or absence of light while downstream neurons progressively piece together an image so that it is subjectively experienced as a whole.
(3) *The nervous system executes hierarchical processing in anatomically segregated neural areas.* The diverse neural circuitries (as outlined in principles 1 and 2) are generated during development under the control of specific gene regulatory networks (Giacomantonio and Goodhill, 2010). To function, these networks must be expressed in anatomically segregated regions of the nervous system.

### Applying Neural Design Principles to Subjective Experience

When we refer to understanding the neuroanatomical basis of subjective experience, we are not simply referring to the identification of specific brain regions that are activated when sensory stimuli are processed. We are instead concerned with identifying the neural computations necessary for subjective experience (principle 1). With respect to the visual system, conversion of light into neural activity within RGNs is necessary for downstream generation of visual experience. However, RGN activity, by itself, is not very informative about downstream subjective experience. A more powerful algorithm for assessing the likelihood that an organism has, at least, the potential for subjective experience is realized by identifying a series of hierarchically ordered neural computations (as per the principles outlined above) that are necessary for subjective experience. Importantly, one need not know the complete pathway in order to begin to assess the likelihood that an animal is capable of subjective experience. Consequently, this approach sidesteps a common roadblock that stymies discussion of subjective experience in animals—i.e., the idea that it is futile to attempt to address animal consciousness when human consciousness remains a mystery.

Given that the cerebral cortex is the site of subjective experience in humans, we should expect that the cortex will contain specific regions (principle 3), that are hierarchically organized (principle 2) and executing neural computations (principle 1) for subjective experience. We differentiate between neural computations that are necessary for generating the contents of experience (e.g., a unitary representation of a car with doors and wheels correctly bound together with appropriate spatial relations and with the correct size and color) from those neural computations generating the actual subjective experience of the stimulus (i.e., perceived as something rather than nothing). The primary visual cortex (V1) is typically necessary for both the normal rich visual experience of something as well as the contents of that experience. Lesions to this brain region can either cause blindness or produce distorted visual content in humans. However, V1 alone is not sufficient for visual subjective experience—it requires top-down feedback from other posterior cortices (Lamme, 2006, 2020). Interestingly, in clinical cases it has been shown that V1 is not strictly necessary for visual subjective experience of simple flashes of light since direct stimulation of higher visual cortex in V1-injured blind patients can elicit such visual experience (Key and Brown, 2018; Mazzi et al., 2019). Thus, subjective experience occurs in higher cortical areas and is dependent on hierarchical processing. While others do agree with the need for hierarchical processing (Boly et al., 2017), they draw the line at including the purported highest level—prefrontal cortex—in subjective experience. This conclusion seems at odds with those theories advocating a role for global integration and is not supported by lesion studies, particularly those in primates that show that the prefrontal cortex is necessary for visual subjective experience (Odegaard et al., 2017).

Removal of large areas of the primate cortex including the dorsal prefrontal cortex, superior temporal cortex, and parietal cortex—while leaving the visual cortex intact—caused immediate blindness (Nakamura and Mishkin, 1986). Interestingly, these lesioned monkeys began to regain visual behavior within months of their operation suggesting that remaining cortical areas were sufficient to reinstate visual function. When the lesion was further expanded to include the ventral-frontal cortex, animals then became permanently blind. This result is inconsistent with theories (Boly et al., 2017; Odegaard et al., 2017; Martín-Signes et al., 2021) that maintain that consciousness arises exclusively in low levels of hierarchical sensory processing streams. As new neuroscientific approaches are being applied, this debate is shifting heavily in favor of the prefrontal cortex playing a fundamental role in determining the level of consciousness (Pal et al., 2018) as well as generating the contents of consciousness (Joglekar et al., 2018; van Vugt et al., 2018; Kapoor et al., 2020; Michel and Morales, 2020; Panagiotaropoulos et al., 2020a). However, there is no need here to engage in a protracted debate about the relationship between the prefrontal cortex and subjective experience (Brown et al., 2019); it is enough to acknowledge that higher cortical areas outside of visual cortex are clearly necessary for experience—a conclusion consistent with hierarchical processing design principles and one that is widely supported by participants on either side of the front-versus-back brain debate of subjective experience. In the next section, we introduce our framework and discuss how it incorporates hierarchical processing using predictive models as the basis for subjective experience.

## A Hierarchical Forward Models Framework for Subjective Experience

### Levels of Awareness Necessary for Subjective Experience

In Section “Terms of Reference” we discussed how awareness was integral to subjective experience but we did not define awareness or explain how it could come to be realized in a nervous system. In general terms, awareness arises in a system when that system possesses information about its own internal state(s). Since the medium of information exchange in nervous systems is neural activity, an internal state is the neural activity in a particular subsystem at any instance. Thus, subjective experience of sensory stimuli is dependent on the brain’s awareness of its neural activity within sensory pathways. This premise is strongly supported by experimental evidence. Modulation of neural activity in either sensory processing streams or brain regions downstream of this processing disrupts subjective experience (Rainville, 2002; Koyama et al., 2005; Coghill, 2010; Ploner et al., 2010; Boly et al., 2017; Odegaard et al., 2017; Key and Brown, 2018).

**FIGURE 1:**
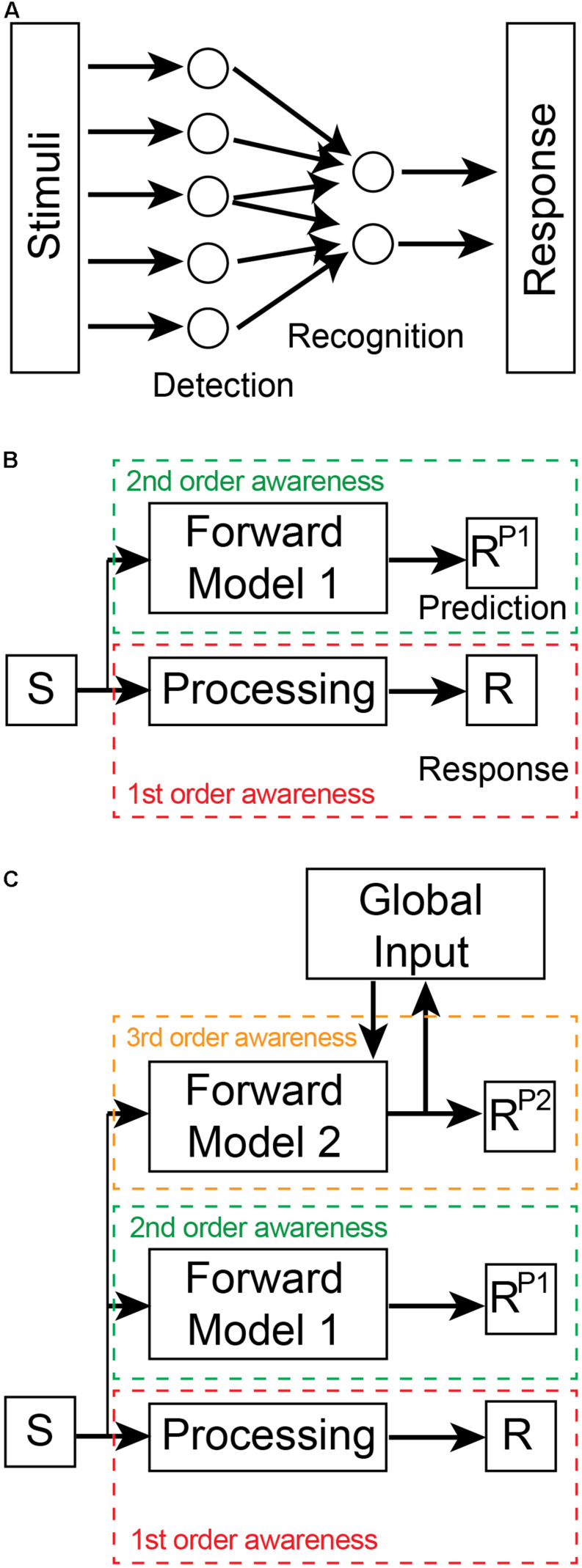
Levels of awareness. **(A)** First-order awareness involves detection and recognition processes within the processing stream from stimulus to response. In this case, recognition involves the convergence and divergence of inputs from detection devices. The recognition devices integrate these inputs which leads to a downstream response. Stimuli are representative of either direct sensory inputs from the environment or of downstream sensory representations. Similarly, the Response could be motor commands or upstream neural representations within the processing stream. This first-order awareness schematic is not meant to represent the entire sensory-motor processing pathway but only a part thereof. **(B)** Second (2nd) order awareness (green dashed box) is generated by a Forward Model 1 that predicts the Response (R) of the first (1st) order awareness (red dashed box) processing stream. The dashed boxes indicate that the two awareness processing streams are in different brain regions. The Forward Model 1 receives a copy of the stimulus input and outputs a prediction of the response (R^*P*1^). **(C)** Third (3rd) order awareness (orange dashed box) involves a distinct forward model (Forward Model 2) that generates a prediction of R^*P*1^. Forward Model 2 receives a copy of the stimulus input as well as global brain inputs in order to generate its prediction (R^*P*2^). These global inputs incorporate context-relevant information into the model. In this way, the implicit awareness inherent in the predictions reflects whole of brain information and ensures that the Response of the first-order processing stream is appropriate for the whole system. R^*P*2^ is then broadcast globally where it leads to conscious awareness. The neural connectivity present between awareness layers is outlined in Figure 2.

In order to become aware, a nervous system requires an appropriate device (i.e., a neural circuit/network) to monitor neural activity in a specific subsystem (Lycan, 1995). This monitoring device plays an essential role in modern physiological theories of consciousness (Cleeremans, 2005; Dehaene et al., 2017; Brown et al., 2019; Panagiotaropoulos et al., 2020b). Awareness in nervous systems is hierarchically organized in levels that span preconscious to conscious states (Brown and Key, 2021). Preconscious awareness levels can be defined by two distinct types of monitoring mechanisms: intrinsic representational generators and extrinsic predictive models. First-order preconscious awareness involves information that is representational, i.e., neural activity that encodes the physiological properties of a sensory stimulus (Vigo, 2011; Ganson, 2020). This awareness is generated by detection and recognition monitoring devices embedded within a sensory pathway (i.e., they are intrinsic and part thereof the pathway; Figure 1A). For example, noxious stimuli cause many different classes of primary sensory neurons to be activated in the mammalian nervous system (Duan et al., 2018; Hill and Bautista, 2020). These sensory neurons are monitoring devices and their neural activity represents information about a recent sensory stimulus. Different classes of primary sensory neurons are more or less responsive to different types of sensory stimulation. Each class preferentially converges onto distinct dorsal horn neurons in the mammalian spinal cord—they too, are intrinsic monitoring devices that begin the process of recognizing specific types of noxious stimuli by integrating the information within the nociceptive pathway (this recognition process continues into the thalamus and the cerebral cortex). This low-level preconscious awareness involving detection and recognition exists only as information within the circuitry of the sensory processing pathway.

A system has higher-order awareness when it “understands” how its processing pathways generate future outputs given current inputs—i.e., when the system has learnt the input-output relationship of its sensory processing. In this way, the system can predict its future internal states. These predictions are generated only by models (Heeger, 2017) known as *forward predictive models* (Miall and Wolpert, 1996) that are extrinsic to the monitored sensory pathway (Figure 1B). Such models predict forward states based on their ability to learn the input-output relationship of the sensory processing pathway being monitored. Second-order, preconscious awareness is therefore generated by forward predictive models that monitor the first-order awareness. Fleming (2020) has also recently recognized the importance of awareness arising from predictions of tiered models, but his idea relates instead to the conscious verbal report of awareness.

Model-based predictions need to be distinguished from model-free predictions employed in reinforcement and associative learning (Smith et al., 2006). To predict (*P*) its future response (*R*^*P*^) based on an input stimulus (*S*), a system can apply a conditional rule-based approach such as, “if *S*, then *R*^*P*^” which uses predetermined stored values of *R*^*P*^ for every value of *S*. This is an example of model-free predictions. These predictions are inflexible, pre-determined quantities and, as such, are equivalent to low-level awareness arising from detection, recognition and selection processes. Model-free predictions have been referred to as “retrospective” predictions (Bach and Dayan, 2017). In contrast, model-based predictions are “prospective” predictions that require knowledge of the *S*— > *R*^*P*^ relationship and can be defined as *R*^*P*^ = *f*(*S*), where *f*(*S*) is an operation that is executed on each value of *S* to generate a new *R*^*P*^ in real time and without the need for stored values. Model-based predictions represent a higher level of awareness in the system and have advantages for achieving system goals in dynamic and noisy environments.

Awareness arising from a single forward predictive model is, by itself, not very informative as to the likelihood that a nervous system is capable of subjective experience. For instance, such models play an important role in motor control in the cerebellum (Hull, 2020; Tanaka et al., 2020), a region of the brain unnecessary for subjective awareness (Arrigoni et al., 2015). Moreover, a single forward predictive model performs poorly in complex dynamic systems (Wolpert and Kawato, 1998). To be an effective predictor and controller of local processes, second-order awareness models need to be driven by local inputs. However, by ignoring information from other subsystems that can influence the local sensory pathway, the model’s predictions are error prone. In engineered systems, the use of multiple independent models that prioritize different inputs can partially overcome error limitations (Murray-Smith and Johansen, 1997; Wolpert and Kawato, 1998). These multiple independent models, however, suffer from the need for extensive organizational control to compensate for the lack of cooperation between models (Narendra and Han, 2012; Narendra, 2016). Processing can be simplified, and performance enhanced by hierarchical tiering of models (Wolpert and Kawato, 1998; Otake et al., 2007; Friston, 2008). We refer to third-order preconscious awareness when a second forward predictive model monitors the primary forward predictive model (Key and Brown, 2018; Figure 1C). By incorporating global information from other neural subsystems as input into the second model, this hierarchical arrangement generates faster and more accurate predictions suitable for both local and global control. The feedback connections of the second model also satisfies what is now commonly thought to be a necessary condition for conscious awareness—global broadcasting of information (Dehaene et al., 2017). The neural architecture required for this hierarchical forward models framework is consistent with the three principles of neural design outlined in Section “Basic Design Features Governing Neural Function”: (i) the framework has functionally-dedicated neural circuits; (ii) these circuits are located in independent but interconnected neural regions; and (iii) these regions are hierarchically organized.

**FIGURE 2:**
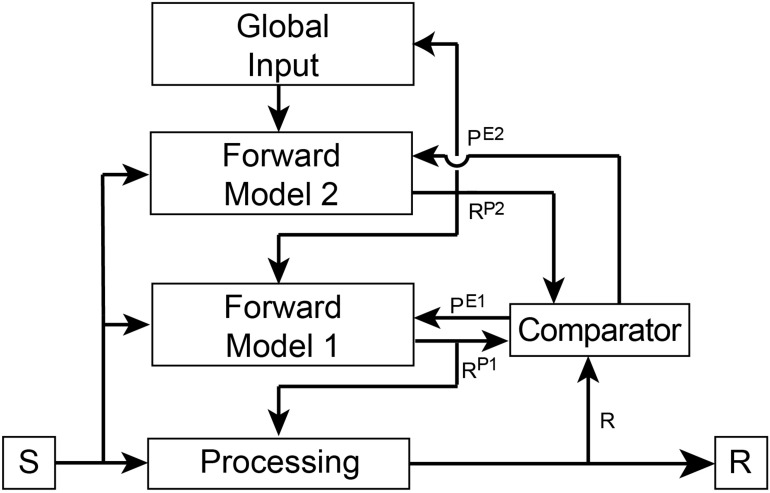
Overview of connectivity of the hierarchical forward models algorithm. The basic architectural layout is as outlined in Figure 1. In this Figure we include an independent Comparator region that performs a simple arithmetic operation of subtraction between its inputs to generate prediction errors (P^*E*1^ and P^*E*2^). These error signals are fed back into the appropriate Forward Model where they update internal parameters to ensure future predictions (R^*P*1^ and R^*P*2^) better match responses (R and R^*P*1^, respectively). The goal of the models is to minimize prediction error. Predictions are also fed back into the lower processing stream to bias ongoing processing toward the predicted response. In this way, a hierarchical top-down control process is created whereby the predictions of Forward Model 1 influence the response of the first-order processing stream and the predictions of Forward Model 2 influence the response of Forward Model 1. This hierarchical processing ensures that the response of the primary processing stream is governed by global states and both forward models. First-order and second-order awareness are therefore simultaneously contributing to control processes as well as to conscious awareness.

Forward predictive models are dependent on a basic architecture to execute *f*(*S*) (Figure 2). Such a model must receive a copy of the input driving the processing pathway. The output of the model is a prediction of the pathway’s output. To ensure accuracy of its predictions, the model’s output is then compared to the output of the first-order awareness pathway within a separate region known as a comparator. The output of the comparator is a prediction error that is then fed back into the forward model to flexibly and adaptively update internal parameters to ensure that future predictions more closely match the future output of the processing pathway. Predictions are also fed back into the processing pathway where they are used to control its internal state and to further ensure the real output matches predicted output. Thus, the second-order awareness circuitry is self-regulating and provides control over the monitored pathway in near real-time. In third-order awareness, another forward model monitors the lower-level forward model to create a hierarchical processing stream.

We contend that the first-, second-, and third-order awareness levels we have outlined above are preconscious states. There is nothing in the circuitry as defined that would indicate that this processing has yet reached conscious awareness and could “feel like something.” However, this hierarchical neural architecture is necessary for conscious awareness since select lesions to the underlying circuitry perturbs subjective awareness of sensory stimuli (Key and Brown, 2018). At this stage, we are not concerned either with the details of the microcircuitry in the various neural regions performing the neural computations or with how these computations are executed. Neither do we intend to define subsequent processing events that enable third-order predictions to become conscious. For our current purpose, it is enough that the hierarchical arrangement of two forward predictive models defines an architecture that is a necessary condition for a nervous system to potentially become consciously aware of its internal states. This conditional requirement is consistent with mounting evidence for the role of hierarchical prediction in subjective experience (Blakemore et al., 2002; Hickok, 2012; Tiippana, 2014; Haggard, 2017).

### Relationship to Other Predictive Coding Algorithms

Our framework is different from predictive coding algorithms that use inference (i.e., predictions) from hierarchical inverse models *embedded within* the internal sensory processing stream to determine the likely cause of the sensory inputs (Friston, 2008; Heeger, 2017). It is debatable whether such predictive processing theories can explain subjective experience since they rely on neural computations that are involved in generating non-conscious sensory content (Marvan and Havlík, 2021). In contrast, our framework uses preconscious awareness states arising from monitoring devices (prediction models) residing outside of the sensory processing stream as the substrate for the awareness of sensory content. Our hierarchical forward model framework has similarities to higher-order theories of consciousness (Lau and Rosenthal, 2011; Brown et al., 2019) in that both involve re-representations of first-order representations of sensory stimuli. For example, Cleeremans’ ‘radical plasticity thesis’ is based on the premise that subjective experience requires knowledge of the brain’s internal states (Cleeremans, 2005, 2011; Cleeremans et al., 2020). He has proposed that a single, higher-order re-representation (i.e., a metarepresentation) of sensory representations by an independent monitoring neural circuit is what provides that knowledge. While Cleeremans has noted that additional processing steps downstream of the metarepresentations are needed to realize subjective experience, he stops short of defining them. In our approach, a second monitoring device (forward prediction model) places awareness of local processing in the context of global events and, in doing so, ensures that awareness is functionally significant and available for the whole system.

We note here that both our hierarchical forward models algorithm as well as predictive coding frameworks seem to be ‘synaptic centric.’ That is, they do not take into account possible influences from neuromodulators (neuropeptides and monoamines) that can act at a distance to modulate neural states. We do not wish to ignore these important influences as they clearly have homeostasis functions, however, their slow timeframe of action is inconsistent with the rapid experience of sensory stimuli such as in pain. The shortest timeframe for modulators is in the order of seconds which is far beyond the millisecond timeframes of synaptic responses needed for rapid sensory processing (van den Pol, 2012).

### The Hierarchical Forward Models Framework in Pain

We have previously demonstrated that the human cerebral cortex possesses the required neural architecture to execute the forward models algorithm with respect to the internal processing of noxious stimuli (Key and Brown, 2018). Not only does the human brain contain the appropriate circuitry required for this framework but the cortical regions are also able to compute the necessary predictions and prediction errors in a temporally appropriate sequence. Moreover, perturbations to the associated circuits and their cortical regions are known to interfere with pain perception in humans.

We contend that any animal lacking the neural architecture to execute the hierarchical forward models algorithm within the context of processing noxious stimuli is incapable of subjectively experiencing pain. It should be remembered that if an animal lacks the architecture necessary for the first forward model (Forward Model 1) they will not be able to be become aware of their internal processing and hence will not be able to subjectively experience that processing. Nonetheless, they will still be able to respond to the sensory stimulus because the basic stimulus-processing pathway (Figure 1) is independent of the model. To be clear, we are not demanding that any animal capable of experiencing pain must possess a cerebral cortex—the burden of proof for such an assertion is extremely high. Rather, we argue that the framework strictly requires the following criteria be met: (1) that independent neural regions must exist to perform the necessary computations (predictions, prediction errors, fine-tuning of models, and prediction-based control of internal states); (2) that these neural regions must be appropriately interconnected by axon pathways; and (3) that hierarchical processing is performed in a temporally appropriate manner. Together, these three criteria provide a test for assessing the likelihood that an animal is, at least, capable of subjectively experiencing pain.

## Do Insects Possess the Neural Architecture Necessary for Pain?

### Generic Structure-Function Relationships in the Fly Nervous System

Given that insects are a diverse and large group of animals within the Arthropoda phylum, we will limit ourselves to discussing only *Drosophila*, as it is the most common insect experimental model in neuroscience. The *Drosophila* nervous system consists of a longitudinally running ventral nerve cord (VNC) located in the ventral thorax and an anterior bulbous brain within the head (Court et al., 2020). For those more familiar with vertebrate nervous systems, the VNC is often likened to the spinal cord. The VNC contains the principal circuitry for executing complex motor behaviors. For example, male flies decapitated while mating will continue copulating and then subsequently groom themselves normally (Crickmore and Vosshall, 2013). The *Drosophila* brain consists of two gross zones—the supraesophageal zone and the subesophageal zone—with each containing an outer layer of neuronal cell bodies (cell body rind) and an inner core rich in neuropil (central brain). The brain consists of 12 supercategories containing 43 units of neuropil regions, many of which are demarcated by encapsulating glial fibers (Ito et al., 2014). The supercategories are referred to as: optic lobe, mushroom body, central complex, lateral complex, ventrolateral neuropils (which includes the posterior lateral protocerebrum), lateral horn, superior neuropils, inferior neuropils, antennal lobe, ventromedial neuropils, paraesophageal neuropils and the gnathal neuropils.

Ascending and descending connections between the brain and VNC course in longitudinal tracts and allow for flexible modulation of VNC-driven motor behaviors by internal brain states. For example, the initiation of mating requires a transient driving input from the brain to the VNC (Zhang et al., 2016). Dopaminergic neurons in several brain regions were found to project to the superior medial protocerebrum within the superior neuropils supercategory where they synapse on ∼40 neurons (called P1 neurons). The activity of dopaminergic inputs to P1 decreases after copulation and it requires several days to return to pre-copulation levels. This decrease in dopamine signaling decreases the responsivity of P1 neurons to female-derived stimulatory inputs. P1 neurons project to and innervate motor command neurons (that control multiple motoneurons associated with specific behaviors; Yoshino et al., 2017; Burgos et al., 2018) in the VNC that initiate mating. Taken together, it seems that P1 neurons gate the driving input to mating initiation. This circuitry clearly demonstrates the important interplay between VNC and brain in regulating motor behaviors. The dopaminergic neurons that stimulate P1 neurons are themselves negatively regulated by copulation-induced feedback inputs ascending from the VNC (Ahmed and Murthy, 2019; Lee and Wu, 2020). Thus, brain and VNC act together in a simple feedforward and feedback circuit to control sex drive.

The role of internal brain states in controlling non-random, flexible types of motor behavior in *Drosophila* is being increasingly recognized (Calhoun et al., 2019). Brain states are able to gate the sensory information reaching the motor command neurons and hence modulate the behavioral outcomes. Thus, the brain gating mechanisms described here play important roles in motor control in *Drosophila*.

### Is It Pain or Nociception in *Drosophila*?

The literature is confusing in terms of nomenclature relating to the sensory processing of noxious stimuli in *Drosophila*. For instance, in a recent review on neuropathic pain in animal models, Calvo et al. (2019) claim that there is “high evolutionary conservation of pain” from *Drosophila* to human. And yet, in the very same article they also state “we are a long way from attributing a perceptive quality such as “pain” to lower organisms.” These mixed messages are all too common. In a study of neuropathic pain in *Drosophila*, Khuong et al. (2019) explicitly conflate the terms nociception and pain when they write “acute pain perception (nociception) evolved more than 500 million years ago.” The authors assert, moreover, that they provide “the first description of long-lasting chronic pain in the fly.” Most studies describing pain in *Drosophila* do so on the basis of the fly’s motor responses to noxious stimuli. As discussed in above sections, attributing subjective experience of feelings, such as pain, to animals on the basis of motor responses to noxious stimuli is deeply flawed.

Our strategy here is to examine the macroscale neural circuitry involved in processing noxious stimuli in *Drosophila* in order to determine whether it is consistent with the hierarchical forward models algorithm that we proposed to underpin subjective experience. The advantage of this approach is that it provides a detailed method, independent of behaviorist assumptions, to assess the potential of an animal to experience pain. In some cases, knowing additional information about the anatomy of the local microcircuitry can provide further insights into the putative function of a brain region with respect to the hierarchical forward models framework. We give two simple examples here. First, if the output neurons of a brain region receive converging inputs from both noxious and olfactory stimuli then those output neurons cannot be said to represent the subjective experience of either stimulus alone. Further downstream processing of that region’s output must occur before the experience of either pain or olfaction could emerge (Ma, 2012; Braz et al., 2014). Such circuit knowledge thereby provides clues as to where subjective experience could be generated in a pathway. Second, if a sensory processing brain region projects a copy of its output to a putative comparator module then that copy must necessarily be an exact duplicate that arises from axon collateral branching of a single population of output projection neurons. If, however, the two outputs arise from separate subpopulations of projection neurons then the putative comparator module would generate erroneous prediction errors. Thus, knowledge of the local output neuron circuitry can lead to a better understanding of the putative function of downstream regions.

We begin our analysis here using *Drosophila* larvae since the small size and stereotypical wiring of the central nervous system in these immature animals makes them well-suited to circuit analysis (Eschbach and Zlatic, 2020). Furthermore, the larval central nervous system has the same gross organization (Meinertzhagen et al., 1998) and the same basic neuronal subclasses (Li et al., 2014) as adult animals but with a considerably smaller neuronal cell number. Early larval brains have an estimated 2,000 neurons (Avalos et al., 2019) compared to ∼100,000 in adults (Scheffer and Meinertzhagen, 2019).

### Nociceptive Processing Pathways in the *Drosophila* Larval VNC

**FIGURE 3:**
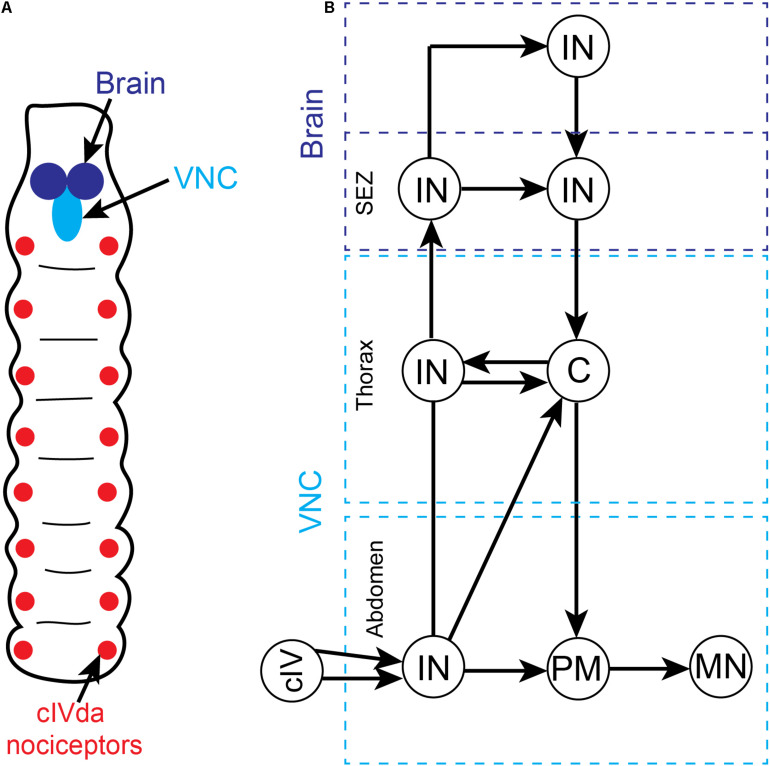
Overview of somatic nociceptive processing in the ventral nerve cord (VNC) of *Drosophila* larvae. **(A)** Schematic depiction of segmentally organized Drosophila larva. The brain and VNC are located in the anterior end of the animal. The sensory neurons that are responsive to noxious somatic stimuli (cIVda nociceptors) are segmentally arranged and project axons into the VNC. **(B)** A simplified circuit diagram of noxious processing pathway and motor outflow in the VNC. While we have not included all the many minor connections, the pathways presented accurately reflect the critical flow of information. There is an ascending sensory pathway involving multiple interneurons at different segmental levels (dashed blue boxes) and a complementary descending motor pathway. Noxious stimuli activate the cIVda nociceptors in the periphery which project into the VNC (light blue dashed boxes) and make contact with segmental interneurons (IN). These interneurons drive local premotor (PM) interneurons which then connect with motor neurons (MN). The segmental interneurons are inter-connected via a series of processing steps that leads into the subesophageal zone (SEZ) and anterior brain regions. Within the descending pathway there are specialized command (C) interneurons in the thoracic segments that control multiple downstream motor neurons.

Late (third-star) larvae display a stereotyped escape response to noxious stimuli consisting of sequential body bending and corkscrew-like rolling (Sulkowski et al., 2011; Chin and Tracey, 2017). These escape behaviors can be elicited by multiple noxious stimuli, including mechanical and thermal stimulation. The principal sensory neurons responsible for detecting these noxious stimuli are segmentally arranged class IV multidendritic (cIVda) whose receptive fields tesselate the larval epidermis (Figure 3A; Chin and Tracey, 2017). When these nociceptive neurons are artificially stimulated or silenced using light-activated optogenetic ion channels, the rolling escape response is induced or eliminated, respectively (Hwang et al., 2007; Honjo et al., 2012; Ohyama et al., 2015). In each body segment (Figure 3B), cIVda sensory neurons converge on second-order interneurons which can either ascend to other segments or directly connect with local premotor interneurons which then connect with local motor neurons. Optogenetic regulation of these interneurons can either induce or reduce rolling behavior (Yoshino et al., 2017). While these segmental nociceptive escape circuits are sufficient to drive motor behaviors, the ascending interneuron projections enable coordination between segmental levels and lead to activation of brain interneurons (see section “Nociceptive Processing Pathways in the *Drosophila* Larval Brain”) that can modulate behaviors via descending pathways (Figure 3B; Ohyama et al., 2015; Kaneko et al., 2017; Yoshino et al., 2017; Burgos et al., 2018; Carreira-Rosario et al., 2018; Hu et al., 2018; Omamiuda-Ishikawa et al., 2020).

The nociceptor escape circuitry in the VNC lacks the architecture required of the hierarchical forward model algorithm for pain (e.g., there is no parallel processing of noxious inputs, no external monitors of the circuit and hence no comparator modules). The connectivity is inconsistent with subjective experience of noxious stimuli, at least in the VNC. Consequently, past claims for pain in insects based upon these behaviors (e.g., Calvo et al., 2019; Abboud et al., 2020; Hehlert et al., 2020; Hesselson et al., 2020) must now be re-evaluated.

Having said this, it is important to emphasize that while the brain is not needed to elicit the escape response, the brain does play a key modulatory role in this behavior. The thoracic VNC interneurons project to the brain where they contact further interneurons. Genetic silencing of select thoracic interneurons reveals that neural processing downstream of these neurons is needed for appropriate escape behavior in response to multimodal sensory cues (i.e., gentle touch and nociception; Ohyama et al., 2015). While the VNC is well suited to drive escape behaviors in response to discrete sensory stimuli, it appears that the brain modulates behaviors in the case of complex sensory cues.

### Nociceptive Processing Pathways in the *Drosophila* Larval Brain

**FIGURE 4:**
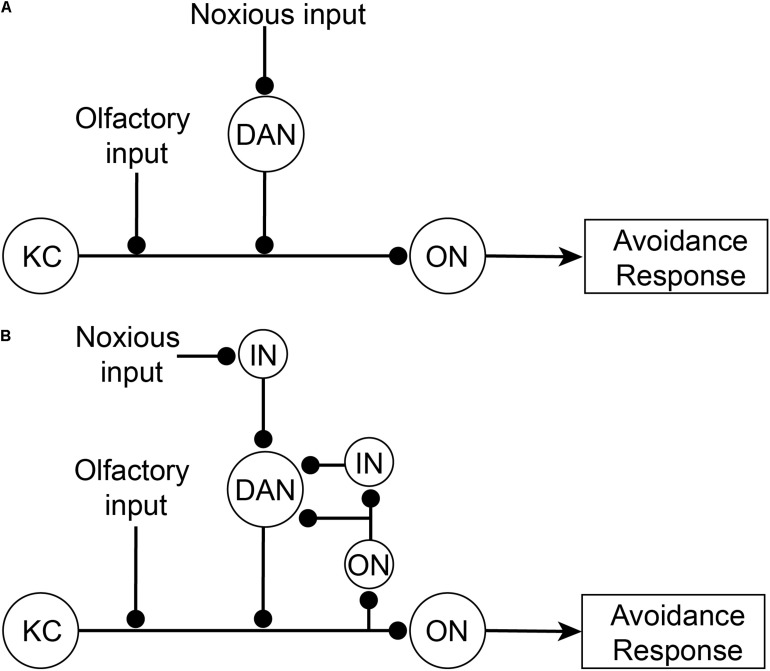
Associative learning circuitry in the Mushroom Body of *Drosophila*. **(A)** Former simplified view of associative learning. The Kenyon Cell (KC) receives dual inputs both directly from olfactory projection neurons in the antennal lobe (see AL, Figure 5A) from a neutral odor as the conditioned stimulus, and indirectly from noxious stimuli via dopaminergic neurons (DAN) in the posterior-lateral protocerebrum (see PLP, Figure 5A). After learning the conditioned stimulus, tThe Kenyon Cells then activate Mushroom Body Output neurons (ON) which subsequently drive downstream avoidance response behaviors. **(B)** Updated circuitry responsible for associative learning. Recently it has been shown that DAN also receive feedback from output neurons (either directly or indirectly via interneurons; IN). DAN act as comparators that compare incoming noxious inputs with Kenyon Cell outputs (via ON) and produce a teaching signal. This teaching signal is used to strengthen olfactory inputs so that after learning olfactory inputs are sufficient, by themselves, to activate Kenyon Cells.

How does the brain modulate the nociceptor escape circuit? Associative learning paradigms provide some answers to this question. *Drosophila* larvae can be classically conditioned whereby they associate an unconditioned noxious stimulus (such as aversive concentrated salt) with a paired neutral stimulus (such as an odor). This conditioning causes the neutral stimulus alone to elicit an aversive behavior (escape) and is dependent on dopaminergic neurons downstream of noxious stimuli that innervate the mushroom body (Schroll et al., 2006; Eschbach et al., 2020b). The mushroom body is the principal higher-order associative learning center in *Drosophila* (De Belle and Heisenberg, 1994; Boto et al., 2020). In a highly simplified interpretation of the canonical circuitry (Figure 4A), the Kenyon cell in the mushroom body receives dual inputs from the olfactory system and dopaminergic neurons carrying information about the conditioned stimulus (olfactory) and the unconditioned stimulus (noxious), respectively. The dopaminergic activity in this circuit was originally proposed to represent the negative value of the noxious stimulus. The output neurons of the mushroom body project downstream to motor pathways to elicit escape behaviors. Following repeated coincident stimulation by olfactory and noxious stimuli, the Kenyon cell is able to drive output neurons and downstream escape behaviors using only conditioned olfactory stimuli. The Kenyon cell is said to have learnt an association between the olfactory and aversive stimuli.

More recently, the dopaminergic neurons have been shown not to directly represent the nociceptive input valence. Instead, they feedforward the difference between inputs from a subpopulation of mushroom body output neurons (either directly or via interneurons) and the nociceptive inputs (Figure 4B) (Eschbach et al., 2020b). Hence, the dopaminergic neurons act as comparators and when there is no difference between signals then the olfactory activity will simply represent the noxious signal. However, if the noxious input differs from the mushroom body output, then there is an error signal that feeds forward to the Kenyon cell to adjust its output to match the altered noxious input (and the system is said to be learning and the dopaminergic activity is the teaching signal). Thus, the activity of the dopaminergic neurons is coincidently driven by recurrent activity from mushroom body neurons representing olfactory inputs and from separate noxious stimuli. Given that dopaminergic neurons represent a mix of two qualitatively different sensory signals, they cannot be said to selectively represent the subjective experience of pain.

The above conclusion is supported by the fact that the underlying circuitry driving associative learning is consistent with that of low-level (non-conscious) detection or recognition. Mushroom body output neurons act like recognition neurons (as represented in Figure 1A) and are simply integrating noxious inputs. Both the mushroom body output neurons and the dopaminergic neurons are embedded in a feedforward-feedback circuit that lacks the structural connectivity to assess the input-output relationship of the whole processing stream, as demanded by the hierarchical forward models algorithm. Taken together, we conclude that the associative learning in larva that drives escape behaviors does not presuppose subjective experience of noxious stimuli. Consequently, there is no reason to believe that the mushroom bodies and associated dopaminergic inputs in *Drosophila* are involved in subjective experience. Associative learning can occur below the threshold of consciousness, and, therefore, it cannot be used as evidence of subjective experience in *Drosophila*. Moreover, when either the mushroom body is ablated (De Belle and Heisenberg, 1994) or when dopaminergic neurons are selectively silenced (Galili et al., 2014) in *Drosophila*, behavioral responses to noxious stimuli remain unaffected.

### Nociceptive Processing Pathways in the Adult *Drosophila* Nervous System

Are there any other noxious processing centers in the *Drosophila* brain that could possibly generate the subjective experience of pain? To address this question, we now turn to the adult fly since recent studies have begun to examine neural correlates of behavior and to place regions of interest within the context of the whole brain connectome. Using adult *Drosophila*, Hu et al. (2018) revealed that the ventral layers of the fan-shaped body, a sensory-motor integration compartment in the central complex (Wolff et al., 2015), were major sites for the processing of noxious stimuli. After demonstrating that fan-shaped body neurons were activated by electric shock to the legs, Hu et al. (2018) used a behavioral choice assay to investigate the function of these neurons in nociception. Animals were given the choice to either enter or avoid an electrified arm of a two-arm chamber. Although the brain is not needed in flies for avoidance of noxious stimuli (Booker and Quinn, 1981), animals do exhibit reduced avoidance behavioal responses to electric shock when a subpopulation of ventral fan body neurons were selectively inhibited (Hu et al., 2018). More importantly, Hu et al. (2018) revealed that optogenetic stimulation of the ventral fan-shaped body neurons was sufficient to cause avoidance in this assay. Given that the fan-shaped body integrates multi-sensory information, it is well suited to contextualize nociceptive inputs and contribute to flexible behavioral responses (Ritzmann et al., 2012). The fan-shaped body is also a major downstream target of mushroom body output neurons (Li et al., 2020; Scaplen et al., 2020), which suggests that it participates in motor control following associative learning. Hu et al. (2018) confirmed this role by showing that inhibiting ventral fan-shaped body neurons perturbed odor-dependent avoidance in animals conditioned to associate an odor with electric shock.

**FIGURE 5:**
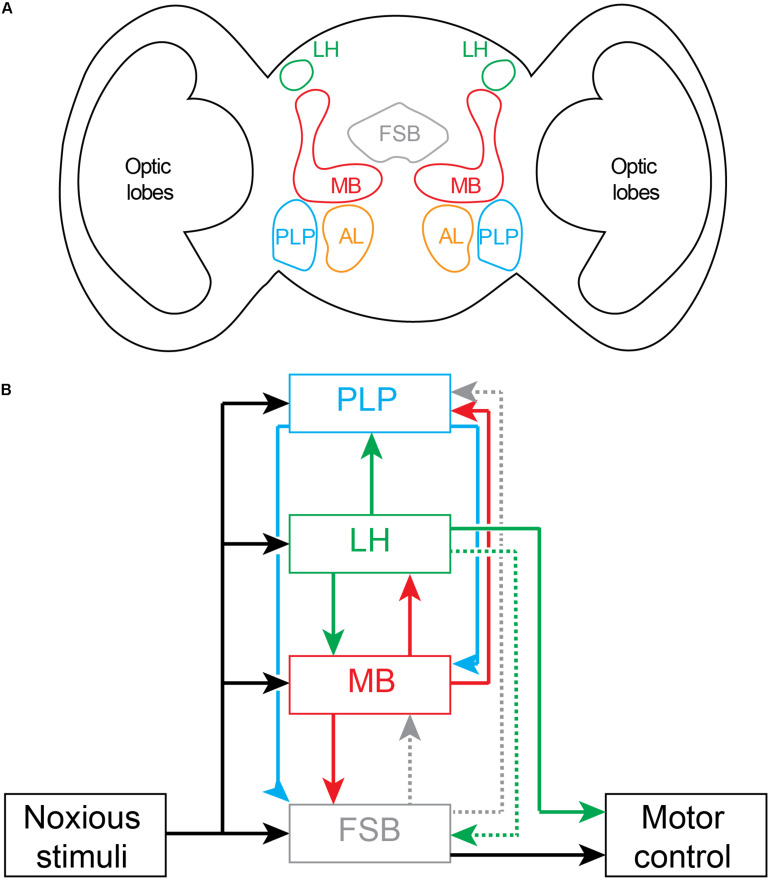
Nociceptive processing in the adult *Drosophila* brain. **(A)** Schematic drawing of coronal section of brain showing location of major noxious processing regions. Fan Shaped Body (FSB); Lateral Horn (LH); Mushroom Body (MB), Posterior Lateral Protocerebrum (PLP); Antennal Lobe (AL). **(B)** Circuit diagram of brain regions processing noxious inputs. Dashed lines represent weak connectivity. The outputs driving motor behaviors arise principally from the fan shaped body and the lateral horn.

What is the role of the fan-shaped body in this avoidance behavior? The associative learning paradigm provides an interesting clue to its function. During associative learning, animals learn to escape an impending noxious stimulus using only the conditioned stimulus as a cue. Because this behavior is then produced in the absence of the noxious stimulus, the fan-shaped body’s role is in motor control rather than in the subjective experience of pain. This conclusion is also consistent with human associative learning paradigms involving electric shock. Subjects do not feel pain while responding to the conditioned stimulus alone (Urcelay and Prével, 2019). The fan-shaped body has limited connections with other brain regions processing noxious stimuli. Two subsets of neurons from the fan-shaped body provide some input to the mushroom bodies and dopaminergic neurons of the posterior lateral protocerebrum (Figure 5, Li et al., 2020). Other connections from the fan-shaped body have recently been mapped, including to parts of the superior and inferior neuropils (Hulse et al., 2020). While an understanding of the connections between brain regions is still emerging, we note that the projection of the fan-shaped body to the mushroom body and to the posterior lateral protocerebrum is not consistent with either of these two regions fulfilling the roles of a comparator module within the hierarchical forward models algorithm. A comparator instead requires the full output of the fan-shaped body in order to generate prediction errors suitable for the feedback correction of predictions. Together, these results suggest that the mushroom and fan-shaped bodies are not involved in generating subjective awareness of noxious stimuli.

In adult flies, aversive (noxious) temperature inputs from the periphery are first transmitted to the terminals of thermosensory projection neurons (also called anterior cells; Galili et al., 2014; Frank et al., 2015) in the posterior antennal lobe. Mapping of subsequent downstream noxious temperature processing has shown that thermosensory projection neurons project sequentially to the mushroom body, lateral horn of the protocerebrum and posterior lateral protocerebrum (Figure 5) (Frank et al., 2015; Marin et al., 2020). These projections to the mushroom body and posterior lateral protocerebrum—the site of noxious-sensitive dopaminergic neurons that project to the mushroom body (Nässel and Elekes, 1992; Claridge-Chang et al., 2009)—are consistent with their role in associative learning involving unconditioned noxious stimuli (as in Figure 4B). One-third of all lateral horn projection neurons receive inputs from the mushroom body output neurons. Lateral horn neurons then project back to the mushroom body where they synapse on output neurons and dopaminergic neurons and modulate teaching signals (Dolan et al., 2019; Otto et al., 2020). Lateral horn neurons also project to lower motor centers to initiate motor behaviors (Dolan et al., 2019). In addition, the lateral horn projects weakly via one neuron type to the dorsal fan-shaped body (Hulse et al., 2020; Lerner et al., 2020). As a site for integration of learned associations and direct sensory inputs, the lateral horn is well placed to control both innate and learned motor behaviors. The role of the lateral horn in innate behaviors has been clearly defined for aversive odors (Seki et al., 2017). There is a dedicated feedforward pathway from peripheral olfactory receptor neurons to projection neurons in the antennal lobe, and then to the ventral-posterior regions of the lateral horn, and from there to specific mushroom body output neurons (Eschbach et al., 2020a). The mushroom output neurons project to the ventral fan-shaped body where they can modulate motor behaviors (Scaplen et al., 2020). While the mushroom body neurons also project to the lateral accessory lobe of the central complex, this region is not activated by noxious stimuli. Taken together, the lateral horn, mushroom body and fan-shaped body form a feedforward pathway for motor control. The processing simply fires back and forth between regions without there being a required site that acts as a comparator of noxious signals capable of building the forward models framework of Figure 1.

The circuitry interconnecting noxious inputs, associative learning and motor pathways in adult flies does not support the hierarchical forward models algorithm. While noxious stimuli are processed in parallel by several brain regions, none of these regions are located external to the processing stream (Figure 5) where they could act as monitors and create models generating higher awareness. Furthermore, none of the regions are appropriately interconnected to serve as comparators in this processing stream. For comparators to operate across this parallel processing, outputs must be equally fed forward into the comparator as well as fed back into lower-level processing streams. None of the outputs from any one of the noxious processing centers in fly brain represents full copies of each other and, hence, the results are inconsistent with the requirements of forward models. Instead, the most likely conclusion is that the circuits underlie low-level (non-conscious) awareness (i.e., detection and recognition events) executing multisensory modulatory control of motor behaviors.

## Concluding Remarks

The argument for the conclusion that insects are not capable of pain defended here should be taken for the presumptive argument it is—its conclusion, although backed by our neuroscientific modeling, remains defeasible. By pinpointing neural processing functions as biomarkers of pain, we avoid appealing to gross similarities between motor behaviors between species, and this allows us to narrow our search down to organisms capable of monitoring their own ongoing internal processes. This awareness, we propose, is generated through internal modeling of the input-output relationships among sensory processing pathways. Such modeling demands a specific type of neural architecture and in its absence, insects will lack subjective experience.

Frameworks like ours that incorporate and recognize the functional constraints of structural interconnectivity on regional brain function are being increasingly being embraced by cognitive scientists (Ito et al., 2020). However, these are yet to be fully appreciated in comparative psychology and philosophy of mind, where the assumption that mental phenomena are or could be ‘multiply realizable’ (Putnam, 1967) has a wide subscription base. Even if one rejects outright the premise that subjective experience requires the hierarchical forward models algorithm, it remains to be explained how insects could possibly generate subjective experience given that there is nothing in their known feedforward-feedback neural circuits, designed as they are to execute motor behaviors, that presupposes consciousness. It is becoming increasingly clear that the more we understand about the neural pathways underlying flexible behaviors in insects, the less likely it is that these behaviors depend on subjective experience (Dvořáček and Kodrík, 2021). Flexible behaviors are readily explained by simple algorithmic rules that transform sensory inputs into motor outputs (Hein et al., 2020; Lee and Wu, 2020). The development of computational models using feedforward neural nets (Faghihi et al., 2017; Springer and Nawrot, 2020) that replicate insect behaviors supposedly dependent on subjective experience (such as first and second-order associative learning) highlights the pitfalls of relying on behavioral biomarkers of insect consciousness. To date, we find no evidence for the insect brain being capable of subjective experience, and the best explanation for this lack of evidence is likely to be the very non-existence of this phenomenon in insects.

## Data Availability Statement

The original contributions generated for this study are included in the article/supplementary material, further inquiries can be directed to the corresponding authors.

## Author Contributions

BK, OZ, and DB contributed to the synthesis and consolidation of ideas as well as the writing of this manuscript. All authors contributed to the article and approved the submitted version.

## Conflict of Interest

The authors declare that the research was conducted in the absence of any commercial or financial relationships that could be construed as a potential conflict of interest.
